# Adapting Real-Time Lung Function Measurements for SARS-CoV-2 Infection Studies in Syrian Hamsters

**DOI:** 10.3390/v16071022

**Published:** 2024-06-25

**Authors:** Rineke de Jong, Wout Nuiten, Albertjan ter Heide, Wilfred Hamstra, Sandra Vreman, Nadia Oreshkova, Katrin E. Wiese, Nora M. Gerhards

**Affiliations:** Wageningen Bioveterinary Research, Houtribweg 39, 8221 RA Lelystad, The Netherlands

**Keywords:** whole-body plethysmography, lung function, Syrian hamster, breathing frequency, SARS-CoV-2

## Abstract

Pulmonary function examinations are critical to assess respiratory disease severity in patients. In preclinical rodent models of viral respiratory infections, however, disease is frequently evaluated based on virological, pathological and/or surrogate clinical parameters, which are not directly associated with lung function. To bridge the gap between preclinical and clinical readouts, we aimed to apply unrestrained whole-body plethysmography (WBP) measurements in a SARS-CoV-2 Syrian hamster challenge model. While WBP measurements are frequently used for preclinical research in mice and rats, results from studies in hamsters are still limited. During unrestrained WBP measurements, we obtained highly variable breathing frequency values outside of the normal physiological range for hamsters. Importantly, we observed that animal movements were recorded as breaths during WBP measurements. By limiting animal movement through either mechanical or chemical restraint, we improved the reliability of the lung function readout and obtained breathing frequencies that correlated with clinical signs when comparing two different variants of SARS-CoV-2 post-inoculation. Simultaneously, however, new sources of experimental variation were introduced by the method of restraint, which demands further optimalization of WBP measurements in Syrian hamsters. We concluded that WBP measurements are a valuable refinement either in combination with video recordings or if average values of measurements lasting several hours are analyzed.

## 1. Introduction

Since the beginning of the COVID-19 pandemic, Syrian hamsters (*Mesocricetus auratus*) have been intensively used as a preclinical model for SARS-CoV-2 infection [1]. Intranasal inoculation of Syrian hamsters with SARS-CoV-2 prototype strain B.1.22 results in reproducible clinical signs, such as weight loss and activity reduction [2]. During peak infection, 4–10 days post-infection (DPI), moderate to severe interstitial pneumonia is observed upon necropsy [2]. Although they provide valuable longitudinal data on, for example, the performance of a vaccine candidate, clinical and virological data are not directly associated with lung function. Lung pathology scores are valuable readouts reflecting lung function; however, these data are based on single, terminal observations and do not allow the development of disease to be followed over time without sacrificing additional animals. In humans, the extent to which lung function is affected is one of the main determinants of COVID-19 severity. COVID-19 patients have a reduced pulmonary diffusing capacity, as well as impaired total lung capacity and residual volumes [3]. Postmortem analyses show features of diffuse alveolar damage with edema, congestion and infiltrates of macrophages and lymphocytes [4]. Likewise, in lungs of hamsters infected with SARS-CoV-2, air exchange appears to be hampered based on histologically observed degeneration of alveolar walls and accumulation of inflammatory cells, edema and hemorrhage. Moreover, respiratory distress is frequently observed in hamsters during peak infection [5]. However, the observation of respiratory distress is a non-numerical parameter that depends on the moment of observation and the experience of the observer and is thereby sensitive to subjectivity. For instance, both a hunched posture as well as sniffing behavior can obscure the accurate visual judgement of breathing in a hamster. To measure lung function in a more objective manner, both invasive and noninvasive methods for small rodents are available (reviewed by Glaab and Braun [6]), and each of these methods is a compromise between accuracy, noninvasiveness and convenience. Unrestrained whole-body plethysmography (WBP) avoids the use of anesthesia, thereby preventing the potential influence on the immune response to a pathogen [7,8], and does not require intubation or implantation of sensors, such as pleural pressor sensors [9]. Repeated measurements of the same animal allow the course of pulmonary disease to be followed over time. While WBP measurements have been investigated more extensively in preclinical research in mice and rats [10,11], studies in hamster models are limited. Published data frequently do not report the breathing frequency (*bf*) of hamsters [12,13,14,15,16]. Instead, the dimensionless parameter enhanced pause (penH), a controversial measure used to interpret bronchoconstriction [17,18,19], is reported, as well as the ratio of peak exploratory flow (Rpef), which is influenced by *bf* and used as a measure of airway obstruction [20]. If *bf* is reported, a decrease post-SARS-CoV-2 inoculation is observed [21]. This is counterintuitive considering that, during pneumonia, an increased *bf* is expected.

Here, we investigated the value of WBP as an additional pulmonary readout during the course of SARS-CoV-2-induced respiratory disease in Syrian hamsters and whether differences between virus strains can be detected. While WBP systems can measure more than twenty different parameters that are partially derived from one another, only *bf* can be independently measured simultaneously by visual observation. As a proof of concept, we therefore focused on obtaining WBP values for *bf* within the physiological range as described in the literature and counted by visual observation.

## 2. Methods

### 2.1. Animals

Two animal experiments were performed under the license numbers 2017.D-0062.005 (4 hamsters) and 2020.D-0007.046 (24 hamsters) at Wageningen Bioveterinary Research in Lelystad, The Netherlands. The pilot study with four hamsters aimed at establishing baseline WBP values for uninfected, healthy hamsters. The second experiment involved 24 hamsters and two SARS-CoV-2 variants and aimed at evaluating whether WBP can be used as a refined clinical readout in SARS-CoV-2 infection studies. In this second experiment, WBP data from 24 hamsters were collected before inoculation. Five hamsters were excluded from the post-challenge analysis due to another exploratory study aim unrelated to plethysmography. Therefore, 19 hamsters were inoculated on D0. Nine hamsters were inoculated with the SARS-CoV-2 prototype strain; four of these hamsters were measured without restraint, and five were measured with chemical restraint. Ten hamsters were inoculated with SARS-CoV-2 strain XBB.1.5, and five animals each were assigned to the unrestrained and the chemically restrained subgroups.

Female Specific Pathogen Free (SPF) Syrian hamsters (*Mesocricetus auratus*), strain RjHan:AURA, were obtained from Janvier (Le Genest-Saint-Isle, France) [2]. The hamsters were 8 weeks of age on the day of arrival. They were housed solitarily according to the most favorable conditions, including a running wheel connected to a rotation counter and cage enrichment. The acclimatization period was 7 days. Body weights were measured regularly throughout both experiments. No uninfected control animals were included in this study because of the risk of accidentally contaminating control animals during handling for WBP measurements and because WBP measurements of each animal were performed before inoculation, which were used as baseline measurements of healthy hamsters.

### 2.2. Mechanical Restraint

Coda^®^ animal holders (‘small rat’ size) (Kent Scientific, Torrington, CT, USA) were manually shortened by several centimeters to fit in the WBP chambers. Each restrainer was assigned to one of the hamsters and provided in the cage as enrichment.

### 2.3. Chemical Restraint

Chemical restraint was performed via an intraperitoneal injection of medetomidine (0.15 mg/kg) applied 5–10 min before the measurement procedures in the WBP chamber. Initially, all chemically restrained animals received atipamezole (0.75 mg/kg) subcutaneously to antagonize the effect of medetomidine. As the animals appeared to develop a tolerance to medetomidine over time and started to recover faster, medetomidine was no longer antagonized after the post-challenge measurements.

### 2.4. Whole-Body Plethysmography

The unrestrained plethysmography setup for rats from EMKA (Paris, France) was used for these studies. All hamsters were allowed to acclimatize to the WBP chambers over the course of several days before the actual measurements. As suggested by the manufacturer, measurements started approximately 10–15 min after the placement of unrestrained animals in WBP chambers and lasted for about 15–20 min per measurement. Hamsters assigned to the mechanical restraint subgroup were placed in Coda^®^ animal holders inside the WBP chambers for increasing durations during the acclimatization period. Measurements of restrained hamsters lasted 5–7 min.

On each of the WBP measurement days, the chambers were first calibrated according to the manufacturer’s recommendations. Respiratory parameters were processed automatically by the acquisition system and iox software (version 2.10.8.25; EMKA Technologies).

### 2.5. Viruses

Passage 2 of SARS-CoV-2/human/NL/Lelystad/2020, lineage B.1.22, was prepared as described previously [2] and is referred to as the ‘prototype strain’. The challenge dose was defined as 10^5^ TCID_50_ on Vero/TMPRSS2 cells. The Omicron variant, hCoV-19/USA/MD-HP40900/2022, lineage XBB.1.5, was obtained from John Hopkins University via BEI resources NR-59104 and passaged once on Calu-3 cells. After sequence verification, passage 2 material was used for challenge at a dose of 10^5^ TCID_50_, as determined on Vero/TMPRSS2 cells. Intranasal inoculation of 100 μL virus stock, diluted in Minimal Essential Medium supplemented with 5% fetal calf serum, 1% antibiotic and antimycotic, 1% L-glutamine and 1% nonessential amino acids (all obtained from Gibco), was performed under general anesthesia as described previously [2].

### 2.6. Postmortem Examination

On day 7 post-SARS-CoV-2 inoculation, all hamsters were euthanized for evaluation of lung pathology as described previously [2].

### 2.7. Data Analysis

Respiratory parameters recorded by the WBP iox software were exported in plain text format. Post-challenge data were trimmed to 5 min. To visualize an entire WBP measurement over time, the PlotTwist web app was used after converting data into tidy format (https://huygens.science.uva.nl/PlotTwist/; [22], accessed on 16 November 2023). The median breathing frequency for each hamster on each measuring day was calculated in Excel (version 2308), and (summary) data were visualized using Graph Pad Prism^®^ (version 10.2.2). For longitudinally measured parameters (relative body weight, relative activity, breathing frequency, PenH and EF50), the total area under the curve (AUC) was calculated, followed by a two-way ANOVA test with a relaxed rule for data normality and variance equality. Group comparisons of relative lung weight and extent of histopathology were performed with one-way ANOVA and a Tukey post hoc test after establishing data normality and equal variances with the Shapiro–Wilk test and the Brown–Forsythe test. Correlation analysis, one- and two-way ANOVA tests, and the tests for normality and equal variances were performed using Graph Pad Prism^®^. Of note, some of the chemically restrained hamsters were still moving during sections of the WBP measurements due to delayed onset of/insufficient sedation. These movements were clearly visible in the individual measurements as an increased recorded breathing frequency. Based on remarks documented on the behavioral observation sheets, a subset of these animals was excluded from the analysis.

## 3. Results

### 3.1. Baseline Measurements of bf Were Highly Variable and Consistently Higher Than the Physiological Range

**Figure 1:**
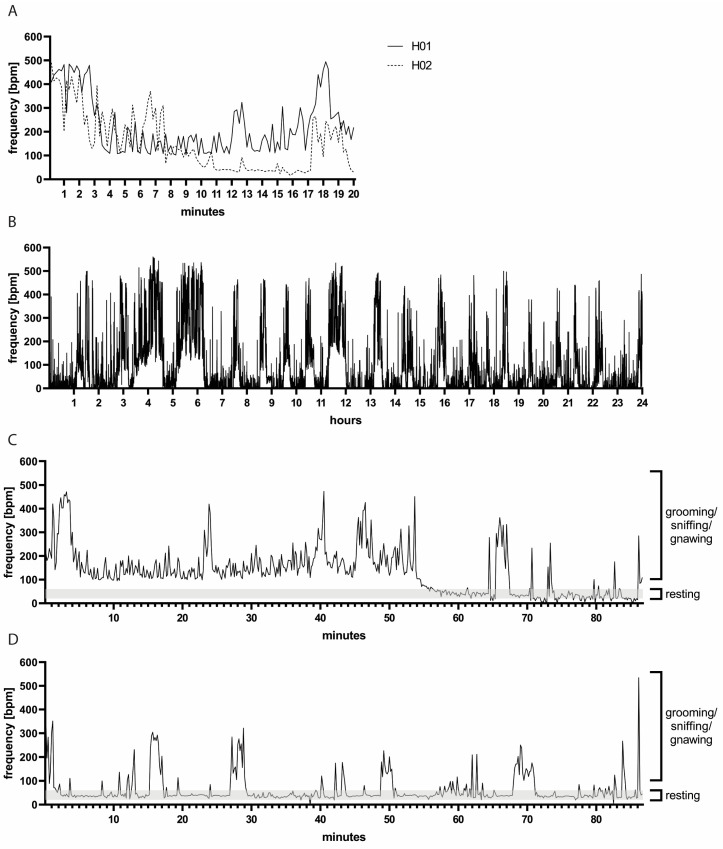
(**A**) Representative *bf* measurements of two hamsters over 20 min. Note the variation between hamsters H01 and H02. (**B**) Representative *bf* measurements of hamster H01 during 24 h. Note the variation in *bf* over time. (**C**,**D**) Measurements of *bf* accompanied by video recording: representative measurements of hamsters H01 and H02, respectively. High *bf* values coincided with hamster movements, such as grooming, sniffing or gnawing, while lower *bf* values were observed during resting periods. Note the variation between the two animals H01 in (**C**) and H02 in (**D**) representative of individual as well as day-to-day variations.

A pilot study to assess baseline measurements in uninfected hamsters revealed highly variable *bf* values (Figure 1A) pronouncedly above the physiological range, which is 25 to 130 breaths per minute (bpm) [23,24,25]. There was clear variation between individual animals (mean *bf* of H01: 224 bpm, range: 101–494 bpm; mean *bf* of H02: 153 bpm, range: 16–490 bpm). We hypothesized that the duration of measurement was insufficient, and thus we examined *bf* over the course of 24 h (Figure 1B). During this extended period, the mean *bf* was indeed within the physiological range (88 bpm). Nevertheless, measurements remained highly variable and unphysiologically high *bf* values above 200 bpm were measured repeatedly.

### 3.2. Unrestrained WBP Measurements Were Confounded by Animal Movement

Next, we aimed at identifying the cause of mean breathing frequencies above the physiological range. We combined WBP measurements with video recordings which were used for manual counting of thoracic movements (Supplementary Video S1). We observed that the hamsters were moving, grooming, sniffing or gnawing during periods when a high *bf* was measured (Figure 1C,D). When the hamsters were at rest, the manually counted *bf* was around 40 bpm (range: 20–60 bpm), and, during these resting periods, the *bf* recorded by WBP was also low (range: approximately 20–80 bpm). Of note, some hamsters moved more than others in the WBP chambers (two individual animals are depicted in Figure 1C,D), and there was also substantial day-to-day variation, further complicating the establishment of a standardized measurement protocol.

### 3.3. Restraining Hamsters for Improved WBP Measurements

**Figure 2:**
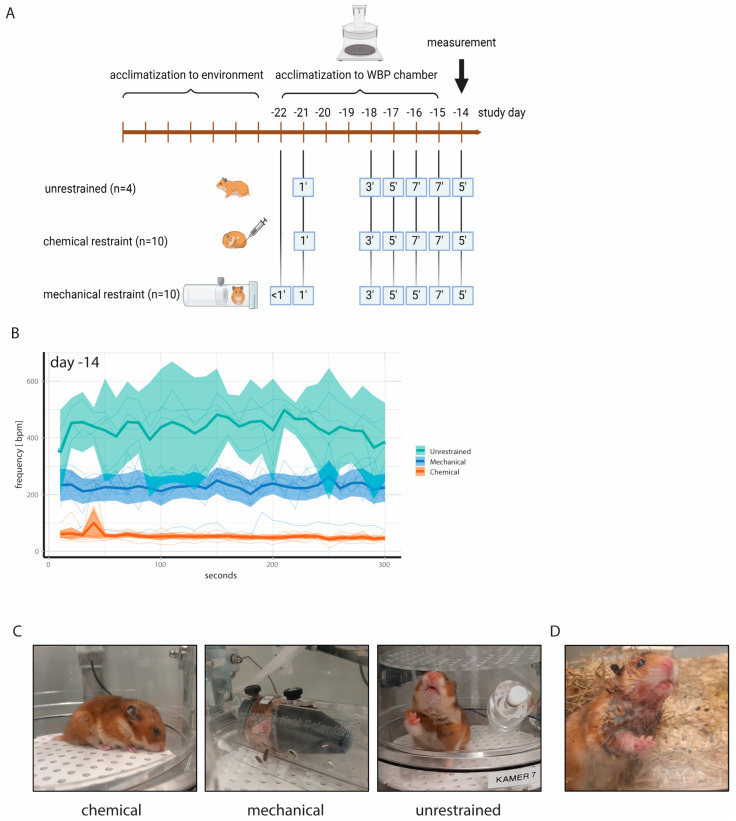
(**A**) Experimental design to acclimatize hamsters to WBP chambers and to animal holders for mechanical restraint. Medetomidine injection was employed as chemical restraint and did not require additional acclimatization other than to the WBP chambers. All animals were allowed to acclimatize to the WBP chambers for increasing time intervals. (**B**) Absolute *bf* values with ranges on study day—14. Note the low *bf* values of chemically restrained hamsters compared to unrestrained hamsters. Thin lines represent individual animals. (**C**) Representative photographs taken during WBP measurements. (**D**) Wet fur of mechanically restrained hamsters due to condensation of exhalations in the animal holder.

While video recordings can aid in identifying reliable WBP measurement windows, associated analysis is laborious and impractical for routine use. We therefore aimed at restricting the movement of the hamsters and thereby enhancing resting periods by either mechanical or chemical restraint. To this end, a second animal experiment involving 24 hamsters was designed. Small rat holders were chosen as mechanical restraints for hamsters due to availability and their being an appropriate size to fit in the WBP chambers. To facilitate acceptance, each hamster was provided with an individual holder which simultaneously served as cage enrichment. By this means, the animals were able to become acquainted with the holders to ensure minimization of stress and anxiety during physical restraint. The hamsters were placed in the holders in the WBP chambers for increasing time periods during a 7-day acclimatization period. Unrestrained control hamsters were allowed to acclimatize to the WBP chambers for similar time periods (Figure 2A). Medetomidine sedation served as a chemical restraint and required no additional acclimatization other than to the WBP chambers. On study day (D) 14, 5 min WBP measurements of *n* = 10 mechanically restrained, *n* = 10 chemically restrained and *n* = 4 unrestrained hamsters were performed. In this study, the unrestrained hamsters had an average *bf* = 446 bpm (95% confidence interval (CI): 370–522), the mechanically restrained hamsters had an average *bf* = 229 bpm (95% CI: 192–266), and the chemically restrained hamsters had an average *bf* = 52 bpm (95% CI: 45–58) (Figure 2B,C). Compared to rats, hamsters have a shorter and less conically shaped head, and we observed that the mechanically restrained hamsters frequently moved their head away from the center of the conical head piece. In such instances, warm exhalations led to condensation within the restrainer, resulting in wet fur (Figure 2D). Additionally, a few animals gnawed on the inside of the restrainer, creating sharp edges that subsequently caused small skin injuries. This occurred on D14, after which no additional WBP measurements were performed using physically restrained hamsters. Moreover, the *bf* values of the chemically restrained hamsters were closest to the physiological range, and therefore we discontinued investigating the mechanical restrainers. Body weights and activity counts remained stable in chemically restrained animals (Supplementary Figures S1 and S2), and no other animal welfare concerns were noted.

### 3.4. WBP Measurements Following SARS-CoV-2 Inoculation

**Figure 3:**
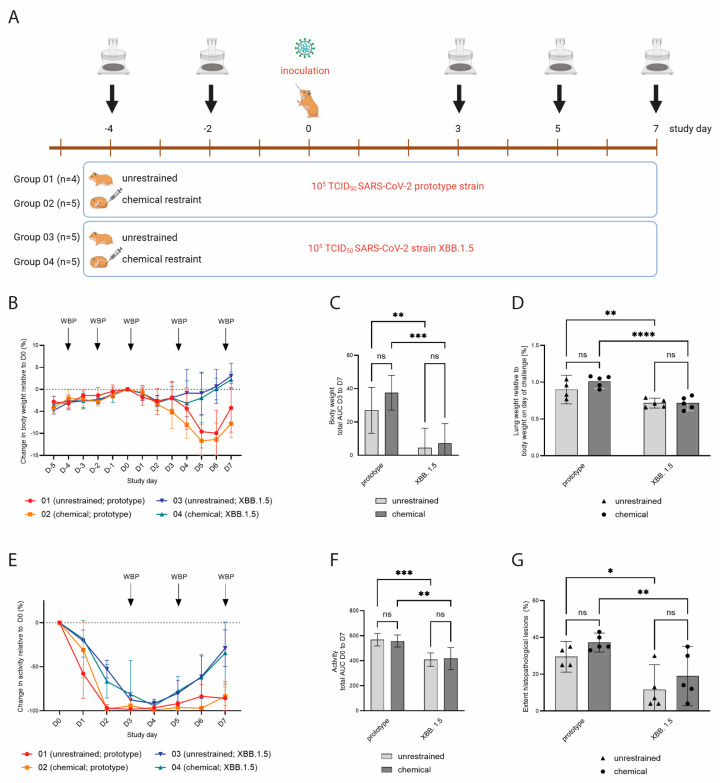
(**A**) Experimental design of a SARS-CoV-2 challenge experiment. Groups 01 (*n* = 4) and 02 (*n* = 5) were challenged with SARS-CoV-2 B.1.22 (prototype strain), and groups 03 (*n* = 5) and 04 (*n* = 5) received SARS-CoV-2 XBB.1.5 (Omicron variant) on study day 0. WBP measurements were performed on indicated study days. (**B**) Body weight change relative to D0, shown as mean values with SDs. Hamsters inoculated with XBB.1.5 showed very mild transient body weight reduction, while hamsters inoculated with the prototype strain had more pronounced and prolonged body weight loss. (**C**) Total area under the curve (AUC) of the body weight change in the period D3 to D7. Error bars represent 95% confidence intervals (CIs). (**D**) Lung weights upon necropsy on D7 relative to body weight on D0. Hamsters inoculated with the prototype strain had higher relative lung weights compared to hamsters inoculated with XBB.1.5. Bars represent mean values, and dots represent individual values. (**E**) Relative activity counts post-inoculation, shown as mean values with SDs. Hamsters inoculated with XBB.1.5 had a faster recovery of activity levels compared to hamsters inoculated with the prototype strain. (**F**) Total AUC of the activity change in the period D0 to D7. Error bars represent 95% CIs. (**G**) Lung histopathology extent, shown as percentage of total left lung lobe. Hamsters inoculated with the prototype strain had a larger affected lung area as compared to XBB.1.5-inoculated hamsters. Bars represent mean values, and dots represent individual values. ns = *p* > 0.05; * = *p* ≤ 0.05; ** = *p* ≤ 0.01; *** = *p* ≤ 0.001; **** = *p* ≤ 0.0001.

**Figure 4:**
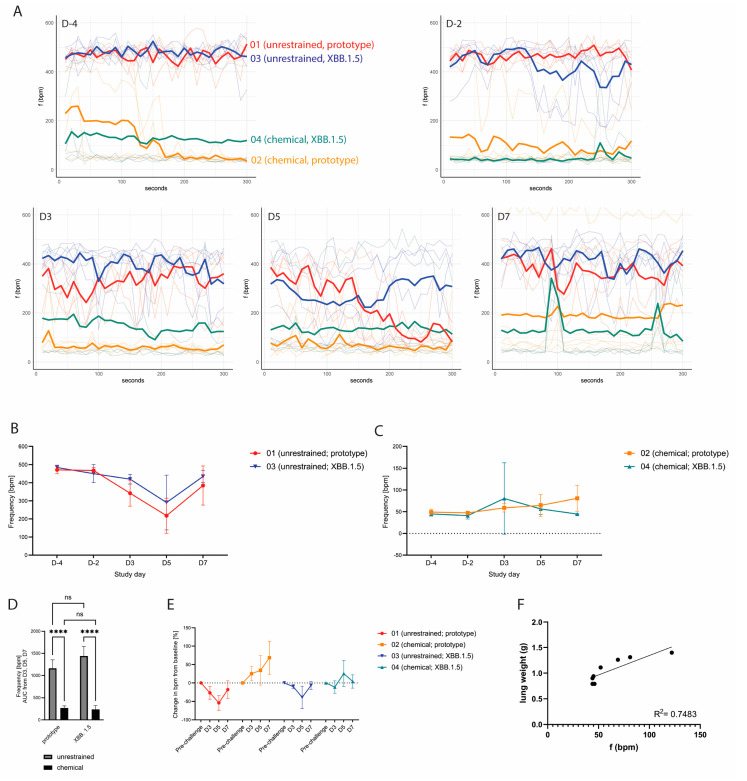
(**A**) Absolute breathing frequencies during 5 min measurements on the indicated study days, shown as individual animals (light colored lines) with the median value per group (bold line). (**B**) Absolute breathing frequencies over time of unrestrained hamsters, shown as mean values with SDs. A more pronounced reduction in *bf* post-challenge was observed for hamsters inoculated with the prototype SARS-CoV-2 strain. (**C**) Absolute breathing frequencies over time of chemically restrained hamsters, shown as mean values with SDs. Post-challenge, chemically restrained hamsters showed a slight increase in *bf*. (**D**) AUC of *bf* measured on D3, D5 and D7. Error bars represent 95% CIs. ns = *p* > 0.05; **** = *p* ≤ 0.0001. (**E**) Change in *bf* in all groups compared to baseline *bf* before challenge, shown as mean values per group with SDs. Chemically restrained hamsters showed a slight increase in *bf* post-challenge, while unrestrained hamsters showed a decrease of *bf* (**F**) Correlation of *bf* values on D7 and absolute postmortem lung weights of chemically restrained hamsters.

We next set out to determine whether WBP measurements in restrained and unrestrained hamsters can be used to detect biologically relevant differences in lung function post-SARS-CoV-2 inoculation. The hamsters were assigned to four groups: groups 01 (*n* = 4) and 02 (*n* = 5) were challenged with SARS-CoV-2 B.1.22 (prototype strain), and groups 03 (*n* = 5) and 04 (*n* = 5) were challenged with SARS-CoV-2 XBB.1.5 (Omicron variant) at a dose of 10^5^ TCID_50_ (Figure 3A). As expected, the hamsters inoculated with the SARS-CoV-2 prototype strain demonstrated more pronounced body weight loss and prolonged reduction in activity compared to the hamsters inoculated with XBB.1.5 (Figure 3B,C,E,F). Relative lung weights were increased upon necropsy on D7 in the prototype-infected hamsters as compared to the XBB.1.5-infected hamsters (Figure 3D). These animals also demonstrated a higher lung histopathology extent (Figure 3G) and severity sum score (Table 1 showing detailed disease severity parameters for chemically restrained hamsters). The differences between the two SARS-CoV-2 strains were significant for all parameters, while no statistically significant change was observed when comparing the unrestrained versus the chemically restrained conditions (Figure 3C,D,F,G). When comparing the effect of virus strain on body weight loss, relative lung weight and histopathology extent, the *p*-value was larger for unrestrained hamsters than for chemically restrained hamsters suggesting that the infection-induced disease is slightly aggravated when repeated chemical restraint is applied. On D4, −2, 3, 5 and 7, WBP measurements were performed under chemical restraint (groups 02 and 04) or without restraint (groups 01 and 03). Of note, although no impact of the chemical restraint on body weight loss had been observed prior to challenge, hamsters that were chemically restrained had slightly increased but nonsignificant body weight loss compared to unrestrained hamsters post-challenge (Figure 3B,C). Consistent with our previous data, unrestrained hamsters had high *bf* values (median: 367–500 bpm) and chemically restrained hamsters had lower *bf* values within the expected physiological range (median: 36–88 bpm) on D4 and −2 (Figure 4A). Hamsters inoculated with the prototype strain had a more pronounced change in *bf* relative to the baseline before challenge compared to hamsters inoculated with XBB.1.5. However, differences in *bf* between the two strains were not statistically significant (Figure 4B: *p* = 0.4333 on D5 and Figure 4C: *p* = 0.0511 on D7, unpaired *t*-test). When evaluating the effect of virus strain versus restraint method on *bf*, clearly, only the latter had a significant effect (Figure 4D). Importantly, on D3 and D5 post-challenge, unrestrained hamsters showed a decreased *bf*, while chemically restrained hamsters had a slightly elevated *bf* (Figure 4E). Besides *bf*, other parameters measured by WBP also differed between restrained and unrestrained hamsters (see Supplementary Figure S3 and Table 1).

### 3.5. Group Discrimination and Correlation of bf Measured by WBP with Clinical Disease and Lung Pathology

We next set out to evaluate whether WBP measurements performed on chemically restrained hamsters were able to discriminate respiratory-associated disease severity between SARS-CoV-2 prototype- and XBB.1.5-infected animals. Three hamsters from group 02 were scored with visible respiratory signs on D7: one hamster was scored with rapid and labored breathing, and two other hamsters were scored with rapid breathing. These three hamsters had an absolute median *bf* of 122 bpm, 69 bpm and 52 bpm, respectively, during the WBP measurement on D7. In contrast, the hamsters in group 04 had median *bf* values between 44 and 46 bpm. Correlation analysis of *bf* on D7 with absolute or relative lung weight of chemically restrained hamsters on D7 revealed R^2^ values of 0.75 (Figure 4E) and 0.65, respectively (Supplementary Figure S4). For body weight change post-challenge, R^2^ was 0.62; other parameters correlated poorly (Supplementary Figure S4).

## 4. Discussion

In this study, we describe challenges of implementing whole-body plethysmography (WBP) measurements in our SARS-CoV-2 Syrian hamster model. Initially, we aimed to implement WBP as a refined clinical readout of respiratory function post-challenge. However, we observed that breathing frequencies measured by WBP were unphysiologically high. Counterintuitively, following challenge, *bf* values were lower than before challenge despite the development of pneumonia. Using video recordings, we observed that sniffing, gnawing, grooming and other animal movements were registered by WBP as breaths. It also became evident that, as opposed to other rodents such as rats, hamsters maintain explorative behavior for prolonged periods of time during WBP measurement despite repeated acclimatization opportunities, and this behavior confounds WBP measurements [26]. Resting periods were observed but were not synchronous between individuals. Video-assisted analysis was suitable to identify time intervals when hamsters were at rest. However, analysis of the video recordings was laborious and only suitable for small numbers of animals. In addition, under biosafety level 3 conditions and with studies requiring a larger number of animals, we consider video-assisted WBP measurements as not feasible for routine use.

There are dedicated devices that facilitate lung function measurements of restrained rodents, mostly rats and mice. Chemical and/or physical restraints are frequently employed, and we aimed at testing both methods in our system. Compared to unrestrained hamsters, the *bf* values measured in mechanically restrained animals (i.e., hamsters fixed inside rodent holders) were lower but still above frequencies described under physiological conditions. In addition, the rat restrainers were not optimally suited for hamsters, and welfare concerns arose during the experiment, despite the fact that the hamsters used the opened restrainers as shelters in their housing cages. Although we cannot entirely rule out that anxiety led to unphysiological *bf* values in the mechanically restrained hamsters, we consider this explanation to be less plausible since the hamsters willingly entered the holders prior to each measurement. During the measurement, however, the hamsters still moved and gnawed inside the restrainers, which could explain the elevated *bf* values. We concluded that the mechanical restrainers tested in this study were neither suitable for hamsters nor sufficient to reduce the movements recorded as *bf* to the physiological range of *bf*. In contrast, chemical restraint by medetomidine led to recorded breathing frequencies within the physiological range and no apparent negative side effects in terms of animal welfare or impaired health under unchallenged conditions. We therefore evaluated only chemical restraint in a subsequent SARS-CoV-2 challenge infection, with unrestrained hamsters serving as controls.

The objective of this challenge study was to investigate whether restrained WBP measurements can be used to detect differences in lung function in Syrian hamsters due to a respiratory infection. To this end, we challenged hamsters with two different SARS-CoV-2 strains, the prototype B.1.22 variant and the more recent Omicron XBB.1.5 variant, as these were expected to cause moderate–severe and mild–moderate disease phenotypes, respectively. Indeed, hamsters inoculated with Omicron XBB.1.5 displayed an overall milder disease phenotype with limited to no body weight loss and faster recovery from activity loss compared to prototype-challenged hamsters. Moreover, hamsters challenged with Omicron XBB.1.5 had lower lung weights and less widespread and less severe pathological changes in lung tissue on D7. It is worth mentioning that the differences between the virus strains were larger in the chemically restrained hamsters compared to the unrestrained hamsters, suggesting that frequent anesthesia increases disease severity, potentially leading to the detection of false significance in differences when comparing two strains. With respect to lung function, the WBP measurements performed pre- and post-challenge corroborated our previous findings that chemical restraint results in physiological *bf* values, in contrast to the observations for the unrestrained control groups. Importantly, relative to the pre-challenge baseline, an increase in *bf* was observed in restrained hamsters after inoculation with the SARS-CoV-2 prototype variant but not in animals challenged with Omicron XBB.1.5. This observation is indicative of more severely impaired lung function in the prototype-infected hamsters, although the difference between the strains regarding breathing frequency was not statistically significant, likely because of the small sample size. The increase in breathing frequencies in the chemically restrained hamsters correlated with more pronounced (histo-)pathological findings and an aggravated clinical picture. In contrast, the hamsters measured in an unrestrained setting and inoculated with either the prototype or Omicron XBB.1.5 showed a prominent decrease in breathing frequency post-challenge that was most likely attributable to a drop in activity and general malaise (i.e., less exploratory behavior). Interestingly, our data on unrestrained hamsters are in line with data reported in the published literature describing a decrease in *bf* and an increase in penH post-SARS-CoV-2 inoculation as enabling discrimination between less and more pathogenic strains [12,14,15,16,21]. While we consider the observed differences between the strains to be real and sufficiently supported by other parameters, we concluded that the presented changes in respiratory data reflect a decrease in animal movements rather than a real decrease in breathing frequency. Thus, our results suggest that the unrestrained WBP measurements in hamsters were strongly confounded by movements and exploratory behavior. Other groups have observed similar challenges in choosing the appropriate time episode for analysis of WBP data collected from unrestrained hamsters [26].

Simultaneously, we also noticed limitations to the usefulness of chemical restraint for WBP measurements. First, it is not possible to include a control to properly assess the effect of medetomidine on lung-function WBP measurements (e.g., absolute breathing frequency). Medetomidine, like many other sedatives and anesthetic drugs, is known to induce respiratory depression [27], which may not only lead to lower *bf* values but also to dyspnea if animals have respiratory disease. Second, the chemical restraint itself turned out to be a source of variation between animals (e.g., differences in depth/length of sedation) as well as for repeated measurements (e.g., development of tolerance over time), which required the exclusion of several measurements from the data analysis. Therefore, a higher number of animals might be needed to detect statistically significant differences between groups with this approach. Third, although the group sizes were small, we noticed a possible trend towards an exacerbated phenotype (increased body weight loss and lung pathology) in restrained versus unrestrained hamsters post-inoculation with the prototype variant, which could be a reason for caution and warrants further investigation. We concluded that other clinical and pathological parameters are more sensitive with respect to the detection of differences between SARS-CoV-2 strains, while, for lung function measurements, a larger sample size would be needed to detect differences. Due to these limitations, for lung function measurements using WBP in hamster challenge studies, we recommend combining unrestrained WBP measurements with video recordings to identify resting periods of hamsters or using average values of WBP measurements lasting several hours.

## Figures and Tables

**Table 1:** Overview of parameters associated with disease severity in chemically restrained hamsters.

Challenge	Hamster ID	Change in bpm on D7	Absolute bpm on D7	Observed Breathing on D7	Max. Body Weight Loss	Body Weight Change on D7	Absolute Body Weight on D7	Lung Weight on D7	Relative Lung Weight on D7 (to Body Weight on D0)	Lung Histopathology on D7	
										Extent	Severity Sum Score
Prototype	B09	+124.0%	122	Rapid + labored	−14.1%	−10.9%	112.6 g	1.40 g	1.08%	35%	11
	B10	+73.3%	81	-	−12.8%	−8.5%	118.8 g	1.31 g	1.07%	43%	11
	B06	+62.4%	69	Rapid	−13.2%	−9.6%	109.7 g	1.26 g	1.05%	40%	10
	B08	+14.4%	52	Rapid	−13.9%	−7.4%	117.4 g	1.11 g	0.90%	35%	10
XBB.1.5	A01	+23.8%	46	-	−4.0%	+3.6	109.9 g	0.79 g	0.66%	12%	4
	A03	+8.6%	44	-	−2.5%	+3.4	120.0 g	0.90 g	0.71%	14%	5
	A02	+4%	45	-	−7.8%	+1.4	116.4 g	0.94 g	0.78%	30%	8
	A05	−20%	44	-	−2.8%	+3.1	122.2 g	0.79 g	0.61%	4%	3

Note: From both groups, one hamster was excluded from this list due to movement/insufficient sedation in the WBP chamber on D7.

## Data Availability

The data presented in this study are available on request from the corresponding author due to (privacy).

## Supplementary Materials

The following supporting information can be downloaded at: https://www.mdpi.com/article/10.3390/v16071022/s1. Figure S1: Body weight change before challenge; Figure S2: Activity counts before challenge; Figure S3: Lung function parameters of (broncho)constriction. Figure S4: Correlation of clinical and pathological parameters with f values measured on D7. Video S1: Video record corresponding to the data presented in Figure 1C,D, https://doi.org/10.5281/zenodo.11422789.
