# Supplementary material for: Adapting Real-Time Lung Function Measurements for SARS-CoV-2 Infection Studies in Syrian Hamsters
Source: Viruses. 2024 Jun 25;16(7):1022. doi: 10.3390/v16071022 (PMC11281489; doi:10.3390/v16071022)
Supplement: Supplementary file 1 [file viruses-16-01022-s001.zip › viruses-3022183-supplementary.pdf]

Supplemental Figure S1

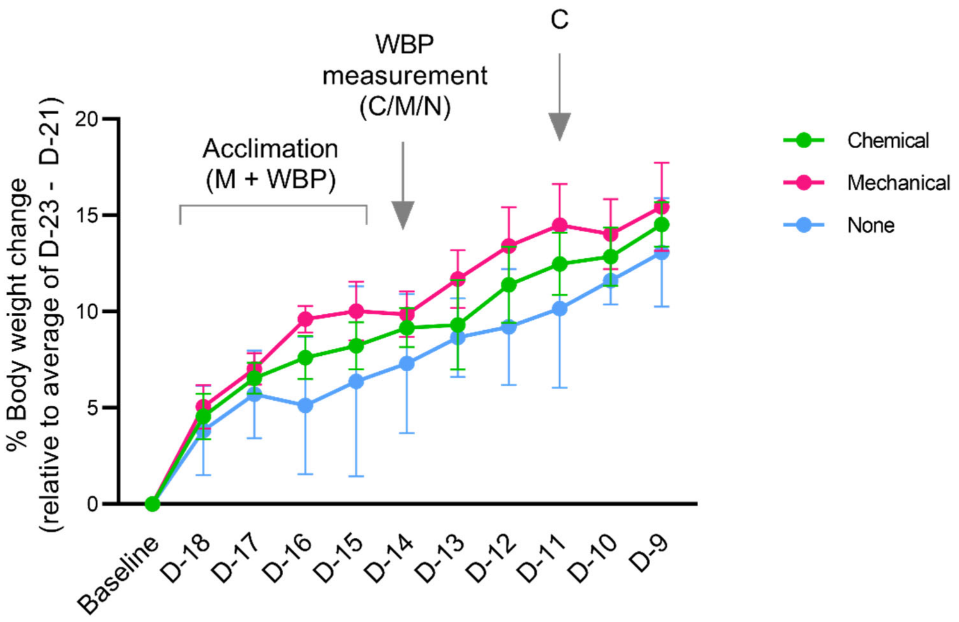

Body weight change before challenge. Change in body weight relative to baseline (average of D-23, D-22, D-21) in %. Symbols indicate the group mean, error bars the 95% CI. During D-18 to D-15, mechanically restrained hamsters were acclimatized to the restrainers and WBP chambers; and unrestrained hamsters were acclimatized to the WBP chambers. On D-14, measurements of all three indicated groups were performed. On D-11, hamsters from the chemically restrained group received an additional sedation to monitor the effect of repeated sedation. M=mechanical, C=chemical, N=none.

Supplemental Figure S2

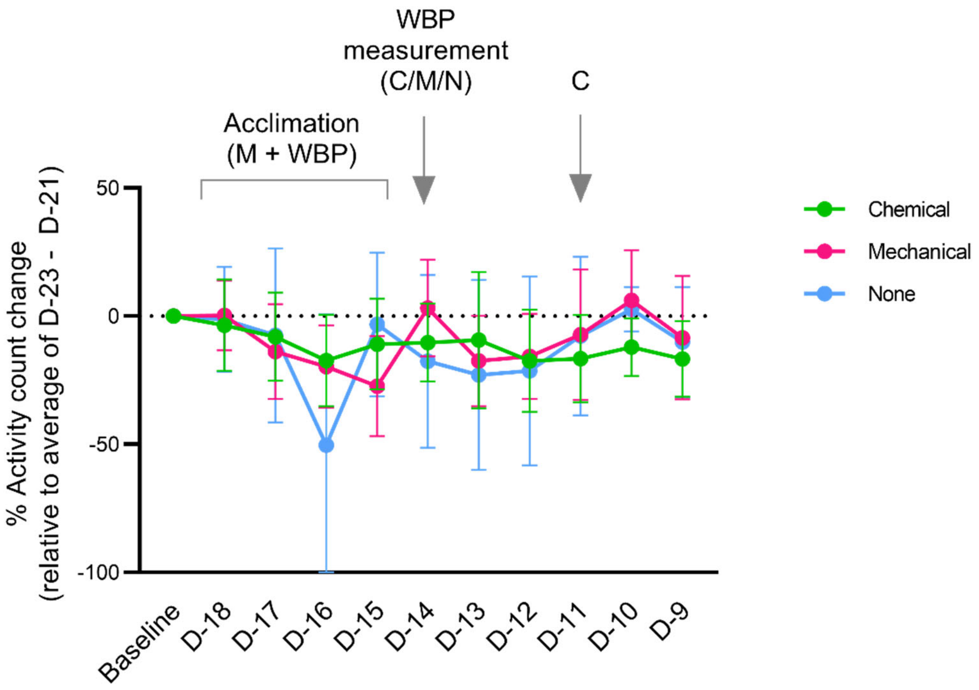

Activity counts before challenge. Change in activity counts relative to baseline (average D-23, D-22, D-21) in %. Symbols indicate the group mean, error bars the SD. During D-18 to D-15, mechanically restrained hamsters were acclimatized to the restrainers and WBP chambers; and unrestrained hamsters were acclimatized to the WBP chambers. On D-14, measurements of all three indicated groups were performed. On D-11, hamsters from the chemically restrained group received an additional sedation to monitor the effect of repeated sedation. M=mechanical, C=chemical, N=none.

Supplemental Figure S3

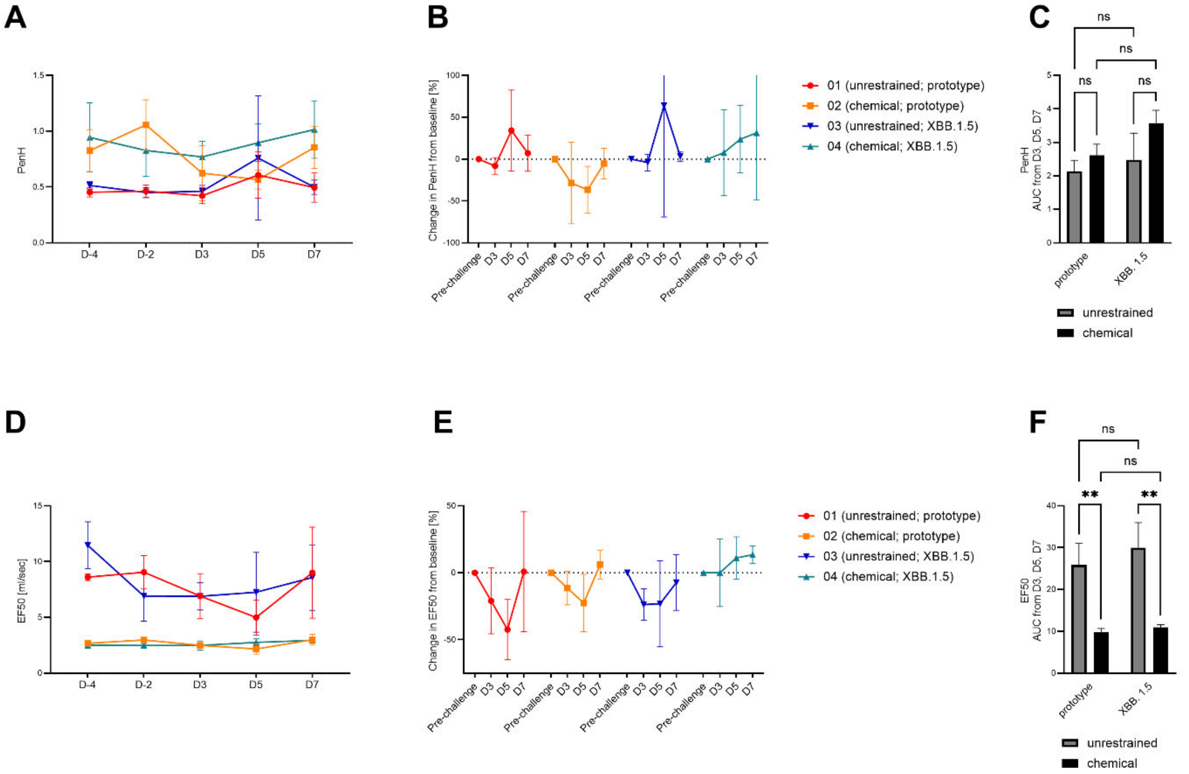

Lung function parameters of (broncho)constriction. **A**) and **B**) PenH: dimensionless (obstruction) index. **C)** AUC of PenH measured on D3, D5 and D7. **D**) and **E**) EF50: expiratory flow at 50% expired volume [ml/sec]. **F**) AUC of EF50 measured on D3, D5 and D7. **A**) and **D**) Absolute PenH/EF50 values per study day. Symbols represent the group mean [based on the median values calculated per 5-minute measurement for each animal], error bars the 95% CI. **B**) and **E**) For each animal, a pre-challenge baseline was calculated. Changes in recorded PenH/EF50 post challenge were calculated relative to this baseline [% change]. Symbols represent the group mean, error bars the SD. **C**) and **F**) Statistical analysis was performed with a two-way ANOVA test. Error bars represent 95% CI. ns= P > 0.05; ****= P ≤ 0.01.

Supplemental Figure S4

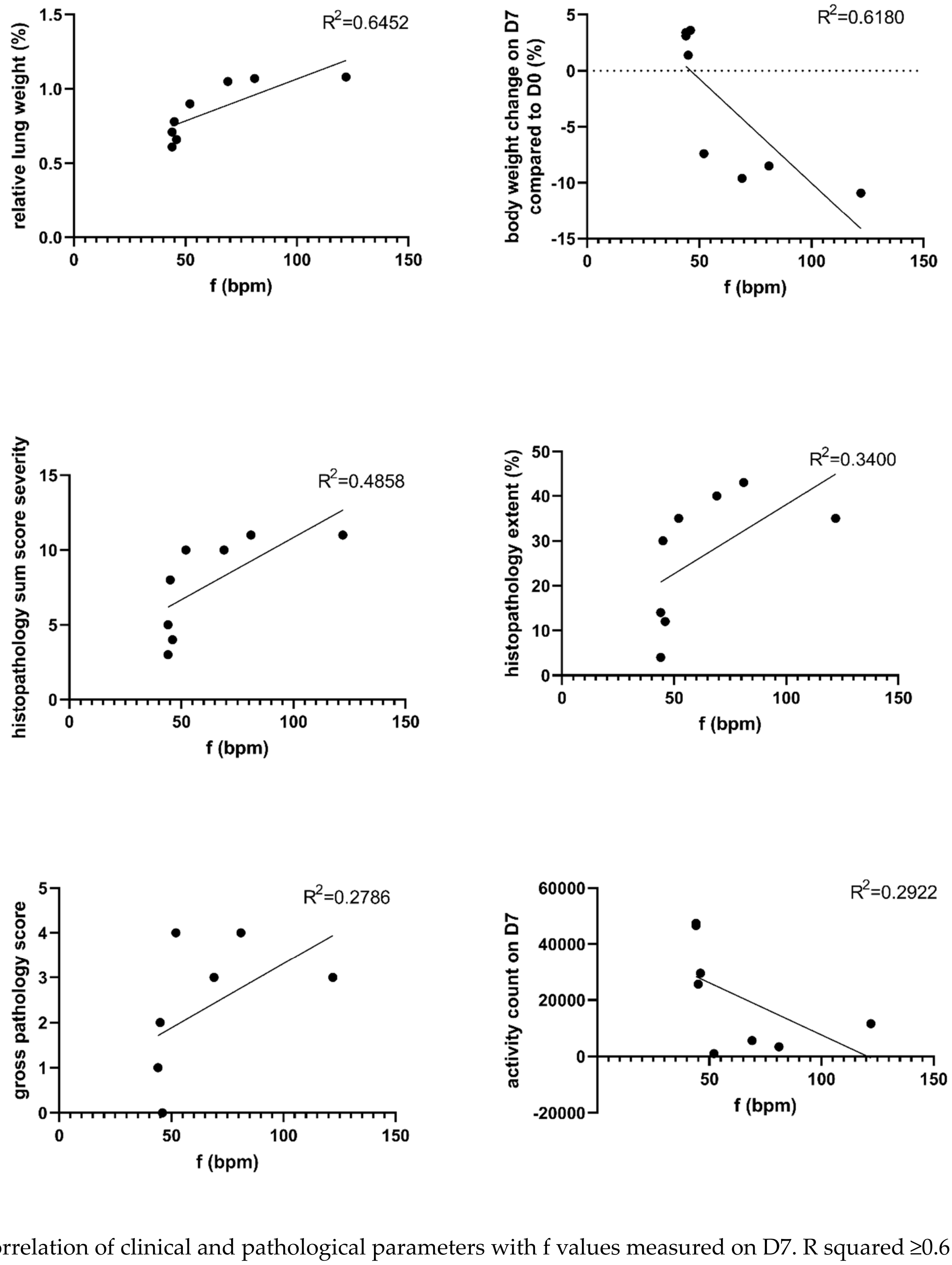

Correlation of clinical and pathological parameters with f values measured on D7. R squared ≥0.6 was observed for relative lung weight and body weight change, while other parameters correlated poorly.
